# Declining Performance on the Qualifying Examination: Modeling of a Potential Inflection Point in Emergency Medicine

**DOI:** 10.1016/j.acepjo.2026.100333

**Published:** 2026-02-12

**Authors:** Suzanne R. White, Michael Gottlieb, Felix K. Ankel, Melissa A. Barton, Yvette Calderon, Susan E. Farrell, Diane L. Gorgas, Lynne M. Holden, Laura Hopson, Oladele Osisami, Mary M. Johnston, Kevin B. Joldersma

**Affiliations:** 1 Department of Emergency Medicine, Wayne State University, Detroit, Michigan, USA; 2 Department of Emergency Medicine, Rush University Medical Center, Chicago, Illinois, USA; 3 American Board of Emergency Medicine, East Lansing, Michigan, USA; 4 Department of Emergency Medicine, Regions Hospital, St. Paul, Minnesota, USA; 5 Department of Emergency Medicine, Icahn School of Medicine, Mount Sinai Health System, New York City, New York, USA; 6 Department of Emergency Medicine, Brigham and Women’s Hospital, Boston, Massachusetts, USA; 7 Department of Emergency Medicine, Ohio State College of Medicine, Columbus, Ohio, USA; 8 Department of Emergency Medicine, Albert Einstein College of Medicine, Bronx, New York, USA; 9 Department of Emergency Medicine, University of Michigan, Ann Arbor, Michigan, USA; 10 Department of Emergency Medicine, New York Presbyterian Hospital, New York, New York, USA

**Keywords:** medical education, board certification, emergency medicine

## Abstract

**Objectives:**

To investigate longitudinal trends in the American Board of Emergency Medicine (ABEM) Qualifying Examination (QE) performance, identify statistically significant inflection points in pass rates, and determine factors associated with the recent decline observed in 2022.

**Methods:**

This was a retrospective, observational study conducted in 2 phases. Phase 1 analyzed all QE attempts from 1980 to 2024 (*n* = 83,254) to identify changepoints using segmented regression and Bayes factor comparisons. Phase 2 focused on QE attempts surrounding the most recent changepoint from 2016 to 2024 (*n* = 23,784) and used multilevel nonlinear piecewise growth models to examine the association between physician characteristics and QE pass rates before and after the 2022 changepoint.

**Results:**

Three statistically significant changepoints were identified: 1988, 2003, and 2022. Although increases in pass rates were noted in 1988 and 2003, 2022 was marked by a decline in pass rates with a steeper drop post-2022 (slope from 2016 to 2022 = −0.17 vs slope 2022 to 2024 = −0.67). From 2016 to 2022, MD graduates had a lesser decline in performance compared with non-MD peers. After 2022, those trained during the COVID pandemic also experienced a lesser decline.

**Conclusion:**

ABEM QE pass rates experienced 3 major inflection points since 1980, with the most recent occurring in 2022 and representing a meaningful decline. Physician-level characteristics, particularly medical school degree type and COVID-era training, were significantly associated with this trend.

**The Bottom Line**

This study sought to identify the presence of inflection points in pass rates and factors associated with the recent decline observed in 2022. Three inflection points were identified, occurring in 1988, 2003, and 2022. Degree type and COVID-era training had the strongest association with the most recent inflection point.

## 1 Introduction

### 1.1 Background

The pass rate on the American Board of Emergency Medicine (ABEM) Qualifying Examination (QE) has declined in recent years, raising concerns about the preparedness of graduates entering independent practice. This phenomenon has raised concern within the emergency medicine (EM) community as this may reflect broader systemic issues within the education of future trainees. Factors that could potentially contribute to this decline include variability in undergraduate medical education,^1^^,^^2^ shifts in the residency applicant pool composition,^3^, ^4^, ^5^ changes in the residency training environment and faculty support,^6^, ^7^, ^8^, ^9^, ^10^ burnout among trainees,^11^, ^12^, ^13^ and rising economic pressures within the health-care system.^14^, ^15^, ^16^ In addition, the COVID-19 pandemic disrupted training experiences at both the undergraduate and graduate medical level.^17^, ^18^, ^19^

### 1.2 Importance

Recent data suggest concerning trends across several domains. EM program directors have reported concerns with residents struggling with clinical reasoning upon entry into residency, including a rise in first-year residents failing to meet expectations for differential diagnosis development, procedural competence, and rapid decision making.^20^, ^21^, ^22^ Although the number of medical schools offering transition-to-residency courses has increased, their content can vary substantially with only a subset offering specialty-specific training.^21^^,^^22^ This gap could leave undergraduate students insufficiently prepared for EM training.^23^ Simultaneously, the total number of EM residency positions has markedly increased over the past decade, introducing greater variability in educational backgrounds and clinical preparation.^4^^,^^24^ Meanwhile, training environments are strained by faculty shortages, financial pressures, and increasing service demands, all of which may compromise resident support and examination readiness.^25^^,^^26^

Prior research has examined select factors associated with individual QE performance in 2017-2019.^27^ However, given the substantial changes since that time period (eg, COVID-19, changes in applicants) and the more recent declines over the past few years, there is a critical need to explore how QE performance compares with prior performances over time to situate the shift in pass rates and to determine which factors are most associated with decline.

### 1.3 Goals of This Investigation

To address these gaps, the present study seeks to determine whether the observed decline in QE performance represents a transient deviation of a persistent trend, whether similar trends have historically occurred, and which specific candidate-, training-, and system-level factors are associated with this decline. By investigating these questions, we aim to better understand the multifaceted challenges facing EM and identify potential strategies for optimizing training and upholding the standards of clinical excellence across the specialty.

## 2 Methods

### 2.1 Study Design

This was a retrospective, observational study investigating the decline in ABEM QE performance among EM certification candidates. The study was conducted in 2 phases. In phase 1, the objective was to determine whether the observed decrease in QE scores over time represents an inflection point vs a single poor testing year. In phase 2, the objective was to explore which individual or environmental characteristics, if any, were associated with the observed decline of QE pass rates in 2022 identified in phase 1. The study was reviewed and determined to be exempt status by the WIRB-Copernicus Group Institutional Review Board. The study adheres to the Strengthening the Reporting of Observational Studies in Epidemiology guidelines.^28^

### 2.2 Setting and Selection of Participants

All QE attempts by EM physicians pursuing ABEM certification were used for this study. This study also includes physicians who sat for the QE (formerly called the Written Examination) and applied to ABEM for certification under the former practice pathway. Physicians who entered through the training pathway were graduates of Accreditation Council for Graduate Medical Education–accredited EM categoric residency programs, combined training programs, or Royal College of Physicians and Surgeons-accredited EM residency programs. Participants for phase 1 included all applicants who sat for the QE from 1980 to 2024. The examination was canceled in 1989, resulting in a missing data point for that year. To explore the recent apparent decline of QE pass rates, participants for phase 2 included all QE attempts made between 2016 and 2024. This window was chosen in an effort to encompass all attempts by physicians taking the QE during the most recent noted decline.

### 2.3 Measurements and Outcomes

The QE is a standardized multiple-choice question examination that assesses EM physicians’ medical knowledge and problem-solving skills. The QE is a secure examination administered annually in the fall. The outcome for the study was a dichotomous variable indicating physicians’ pass/fail status on the QE.

The QE is the first assessment in ABEM’s certification process. Passing it qualifies a physician to take the Oral Certification Examination. The QE reports a scaled score from 0 to 100 and an outcome of pass or fail. The QE is psychometrically equated annually to ensure the reported scaled score maintains meaning, interpretation, and difficulty over time. Its passing score has been approved by the ABEM Board of Directors and is routinely and systematically reviewed and updated through a formal standard-setting process known as the modified Angoff process.^29^ Because each time a new passing standard is established, it creates a new scaled score, this study elected to focus on pass and fail outcomes alone, which remains an objective measure and decision point of meeting the standards of certification for this assessment.

Prior to 2022, physicians’ demographic data were collected from residency programs via the annual ABEM Residency Training Information Survey. Beginning in 2022, physicians’ demographic data were collected directly from physicians themselves via ABEM’s online portal and from responding to a postexamination survey administered after the In-Training Examination (ITE). Physician demographic information included gender (male, female, or not reported), medical school location (United States medical graduates [USMG] vs international medical graduates [IMG]), location of birth (United States vs elsewhere), medical-degree type (MD, DO, or MB), ITE score from their final year of residency training, duration of categoric residency program from which the physician graduated (3 years vs 4 years), and race/ethnicity. Physicians were coded as underrepresented in medicine (URiM) if they identified as American Indian or Alaskan Native; Black/African American; Hispanic, Latino, or of Spanish origin; Mexican American; Puerto Rican; other; or unknown. Physicians who were White or Asian or Pacific Islander were coded as non-URiM. Although the Association of American Medical Colleges’ definition of URiM includes Pacific Islander while excluding Asian, the database contains historical data in which these 2 populations were comingled.^30^ In addition, indicator variables were also created to identify physicians who were in residency training at any point during the COVID pandemic (defined as years 2020-2024), took a leave of absence during residency, or transferred programs during residency.

### 2.4 Data Analysis

Descriptive statistics for all variables of interest for phase 1 and 2 samples were calculated. In phase 1, trend analysis was employed to assess changes in test performance over time to identify changepoints. In phase 2, multilevel nonlinear growth models were used to assess whether any of the selected covariates were associated with the trends identified by the changepoints. Statistical analyses for phase 1 were conducted in R version 4.5.1 (The R Foundation) and in SAS 9.4 (SAS Institute Inc) for phase 2.

#### 2.4.1 Phase 1 analysis

First, the time series of pass rates was visually inspected to provide a starting point for algorithmic identification of possible changepoints. We used segmented regression as the algorithmic approach to detect inflection points (changepoints) in a time series by iteratively splitting the data into segments.^31^^,^^32^ This step was initially performed by starting with the entire time series. The time series was then segmented and, at each step, evaluated to identify the single best changepoint that split the current segment into 2 parts. Methodologically, this can be performed by maximizing a measure of between-segment variance or minimizing within-segment variance, either of which will achieve similar outcomes. We chose to minimize within-segment variance as there is no literature to indicate this would lead to different outcomes. The segmentation process was applied recursively to the newly created segments until a user-specified number of significant changepoints were found. Following the initial application of the algorithm, competing models with different numbers of changepoints were created.

The fit of these models was then compared using a Bayes factor to determine which of the competing models best explained the time series of examination pass rates.^33^ The Bayes factor is the ratio of predictive adequacies of 2 models and represents the degree to which the data can be used to update the relative plausibility of the competing hypotheses.^34^ To compare our models, we calculated this ratio between each of the competing models. The ratio evaluates the probability of H0 (null hypothesis) and H1 (the alternative hypothesis) and determines which outcome is more likely supported by the data (Fig S1).^35^^,^^36^

#### 2.4.2 Phase 2 analysis

In phase 2, multilevel nonlinear piecewise growth models with a logit link function were used to examine the relationship between the log-odds of passing the QE with multiple physician characteristics. Conceptually, examination attempts (level 1) were nested within physicians (level 2). This approach was selected because physicians varied in the number of times they took the QE, and multilevel models can account for differences both within and between physicians, as well as missing and unbalanced data.^37^ Nonlinear models were used because the outcome (ie, passing the QE) was a dichotomous outcome.^37^^,^^38^ Finally, piecewise growth models were used to model the pattern of change in QE pass rates around 2022 that was identified in phase 1. Specifically, 2 piecewise slopes were included in the growth model: 1 represented the trajectory of QE pass rates between 2016 and 2022, and a second represented the trajectory of QE pass rates between 2022 and 2024. The 2 piecewise time slopes were coded so that the intercept of the model reflected the log-odds of passing the QE in 2022 (Table S1).^38^

The continuous variable predictor (ITE score) was grand-mean centered, whereas the remaining predictors were dichotomously coded: type of medical degree (MD = 1), gender (male = 1), race/ethnicity (URiM = 1), country of birth (US born = 1), country of medical school (USMG = 1), length of categoric-residency program (3-year program = 1), receiving training during the COVID pandemic (COVID = 1), transferring residency programs (transfer = 1), and taking a leave of absence during residency (LOA = 1). Although the length of the training format might be considered a program characteristic, we chose to treat this as a physician-level factor because, while a program may change its training length, the residency training length for an individual physician remains the same.

Given that this study was exploratory in nature, a model-building approach was used whereby a series of multilevel nonlinear piecewise growth models were estimated, where QE attempts (level 1) were conceptualized as being nested within physicians (level 2).^37^^,^^38^ First, an unconditional model without any predictors was estimated. Subsequent models iteratively added the 2 time slopes and physician characteristics as fixed effects and random effects, as well as an interaction between each physician characteristic with both time slopes. Model fit was evaluated by examining model deviance, Akaike’s Information Criterion, and Bayes Information Criterion. Likelihood ratio tests were used to compare models.^37^ To aid in interpretation, results of the final model were transformed from log-odds into odds ratios and probabilities. Given the large sample size, a conservative criterion of *P* < .01 was used to guard against type 1 errors.

## 3 Results

Between 1980 and 2024, 65,963 physicians took the QE 83,254 times. On average, physicians took the QE 1.26 times (SD = 1.05). The average pass rate across all years and all attempts was 76%. Demographic characteristics for the full sample are presented in Table 1.

**Table 1:** Physician characteristics for the full and reduced samples.

Demographics	Phase 1: full sample *N* = 65,963		Phase 2: reduced sample *N* = 21,750	
	*N*	(%)	*N*	(%)
Gender				
Female	17,938	(27.19)	8057	(37.04)
Male	35,977	(54.54)	13,658	(62.80)
Missing	12,048	(18.26)	35	(0.16)
Race/ethnicity				
American Indian or Alaskan Native	169	(0.26)	74	(0.34)
Asian or Pacific Islander	5576	(8.45)	2775	(12.76)
Black/African American	2400	(3.64)	1057	(4.86)
Hispanic, Latino, or of Spanish origin	1366	(2.07)	762	(3.50)
Mexican American	521	(0.79)	220	(1.01)
Other	1669	(2.53)	807	(3.71)
Puerto Rican	481	(0.73)	218	(1.00)
Unknown	2173	(3.29)	1748	(8.04)
White	35,044	(53.13)	13,879	(63.81)
Missing	16,564	(25.11)	210	(0.97)
URiM				
URiM	8779	(13.31)	4886	(22.46)
Not URiM	40,620	(61.58)	16,654	(76.57)
Missing	16,564	(25.11)	210	(0.97)
Degree type				
MD	57,138	(86.62)	16,538	(76.04)
DO	8495	(12.88)	5124	(23.56)
Other degrees	330	(0.50)	88	(0.40)
Birthplace				
US born	57,232	(86.76)	19,172	(88.15)
Non-US born	6312	(9.57)	2018	(9.28)
Missing	2419	(3.67)	35	(2.57)
Location of medical school				
USMG	60,570	(91.82)	20,395	(93.77)
IMG	5129	(7.78)	1191	(5.48)
Missing	264	(0.40)	164	(0.75)
Pathway to certification				
Trained	55,596	(84.28)	21,732	(99.92)
Practice	10,163	(15.41)	18	(0.08)
Other	204	(0.31)	--	--
Length of categoric-residency program				
3 y	40,579	(74.51)	15,751	(73.92)
4 y	13,885	(25.49)	5557	(26.08)
Attended residency during the COVID-19 pandemic (2020-2024)				
Yes	13,009	(23.40)	12,957	(59.62)
No	42,587	(76.60)	9023	(40.38)
Leave of absence during residency				
Yes	1346	(2.42)	882	(4.06)
No	54,250	(97.58)	20,850	(95.94)
Transferred during residency				
Yes	1384	(2.49)	564	(2.60)
No	54,212	(97.51)	21,168	(97.40)

DO, doctor of osteopathic medicine; IMG, international medical graduate; MD, doctor of allopathic medicine; URiM, underrepresented in medicine; US, United States; USMG, United States medical graduate.

**Figure 1:**
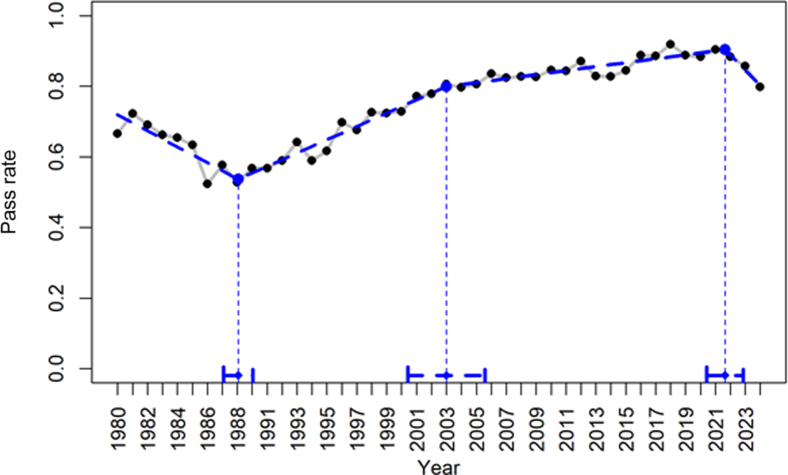
Three competing models to detect changes in QE performance trends. Pass rates by year (gray) and a comparison of 2-(black), 3-(blue), and 4-(red) changepoint models with 95% confidence intervals.

When evaluating the period from 1980 to 2024, at least 2 inflection points are seen visually, with the first change in trajectory appearing between 1985 and 1990, and the second changepoint beginning between 2019 and 2023 (Fig S2). To statistically assess this finding, 3 competing models were then evaluated, consisting of a 2-, 3-, and 4-changepoint model. The 3 models are summarized in Table S2. We then calculated a Bayes factor for each model. The Bayes factor for 2- vs 3-changepoint was 379 (extreme evidence), 2- vs 4-changepoint was 8 (moderate evidence), and 3- vs 4-changepoint was 0.020 (extreme evidence). Therefore, the strongest evidence supported a 3-changepoint model, presented in Figure 1, which included changepoints located at 1988, 2003, and 2022.

Because the recent decline in QE pass rates contained was confirmed as a changepoint (year 2022), we proceeded to phase 2. The purpose of phase 2 was to investigate what characteristics were related to passing the QE before and after the detected changepoint. To that end, for phase 2, the data were restricted to all QE attempts made between 2016 and 2024. This asymmetrical range of years was selected to ensure any physician whose board eligibility period overlapped with the changepoint was included. A total of 21,750 physicians took the QE 23,784 times between 2016 and 2024. In this reduced sample, physicians took the QE a mean of 1.09 (SD = 0.39) times. The overall QE pass rate across all attempts made between 2016 and 2024 was 88.0%. The mean QE pass rate and total number of attempts per year are shown in Table S3. A total of 20,180 physicians (93%) took the QE once between 2016 and 2024.

Results of the unconditional model showed the average log-odds of physicians passing the QE was 3.07; the corresponding average probability of physicians passing the QE was 0.96. The extent to which physicians differed from the average log-odds of passing the QE was 2.79. Using this level 2 intercept variance, 95% of the sample was expected to have predicted QE pass rates ranging from 45% to 100%. The unconditional model yielded an intraclass correlation coefficient (ICC) of 0.46, indicating that 46% of the variability in QE pass rates was attributable to differences among physicians. The ICC was calculated using an approximate level 1 error variance based on the assumption that the level 1 error variance has a standard logistic distribution with a mean of 0 and variance of *π*^2^/3. Using this formula, the ICC was calculated as *τ*_00_/(*τ*_00_+*π*^2^⁄3), in which *π*^2^/3 and *τ*_00_ are levels 1 and 2 error variances, respectively. The substantial ICC supported the use of multilevel models to analyze the data. In subsequent model testing, both time slopes (2016-2022 and 2022-2024) were retained as fixed effects. Neither time slope had significant random effects, suggesting that the rate of change in QE pass rates over time was consistent across physicians.

The final model included both time slopes as significant predictors (Table 2). Both slopes were negative, indicating a general decline in the log-odds of passing the QE over time, with a steeper decline observed from 2022 to 2024 (−0.67) compared with 2016 to 2022 (−0.17). Significant physician and environmental characteristics retained in the final model included medical-degree type, URiM status, medical school location, ITE scores, and completing a 3-year residency. Controlling for other variables, the log-odds of passing the QE increased when physicians graduated with an MD degree, graduated from a medical school in the United States, and scored higher on the ITE. Conversely, the log-odds of passing the QE decreased for URiM physicians and graduates of 3–year categoric-residency programs. Nonsignificant variables and interactions (eg, gender, gender by time slope 2016-2022) were removed.

**Table 2:** Multilevel nonlinear piecewise growth model results predicting the log-odds of passing the QE.

Demographics	Unconditional model		Final model			Prob
	Est (SE)	*P*	Est (SE)	*P*	OR (95% CI)	
Fixed effects						
Intercept	3.07 (0.06)^a^	<.0001	1.94 (0.18)^a^	<.0001		
Time slope 2016-2022			−0.17 (0.04)^a^	<.0001	0.84 (0.78, 0.91)	0.87
Time slope 2022-2024			−0.67 (0.20)^a^	.001	0.51 (0.31, 0.71)	0.46
Degree (MD)			0.86 (0.07)^a^	<.0001	2.37 (2.04, 2.70)	0.34
Degree (MD)∗time slope 2016-2022			0.09 (0.03)^a^	.005	1.09 (1.03, 1.16)	0.70
URiM			−0.42 (0.06)^a^	<.0001	0.66 (0.58, 0.73)	0.52
USMG			0.63 (0.10)^a^	<.0001	1.88 (1.52, 2.24)	0.40
ITE score			0.21 (0.00)^a^	<.0001	1.23 (1.22, 1.24)	0.65
3–year residency program			−0.28 (0.06)^a^	<.0001	0.75 (0.66, 0.85)	0.55
COVID-19			0.28 (0.11)	.012	1.33 (1.03, 1.62)	0.43
COVID-19∗time slope 2022-2024			0.63 (0.20)∗	.002	1.87 (1.13, 2.62)	0.57
Error variance						
Level-2 intercept variance	2.79 (0.18)^a^	<.0001	0.50 (0.09)^a^	<.0001		
Model fit						
Total no. of parameters	2		12			
Deviance	16,963		11,988			
AIC	16,967		12,012			
BIC	16,983		12,108			

AIC, Akaike’s information criterion; BIC, Bayes Information Criterion; COVID-19, trained during the COVID-19 pandemic; Est, log-odds estimate; ITE, In-Training Examination; MD, doctor of allopathic medicine; OR, odds ratio; Prob, probabilities; QE, Qualifying Examination; SE, standard error; URiM, underrepresented in medicine; USMG, US medical graduate.

Note. The ITE score is grand-mean centered. The intercept reflects the log-odds of passing the QE in 2022.

a *P* < .01.

**Figure 2:**
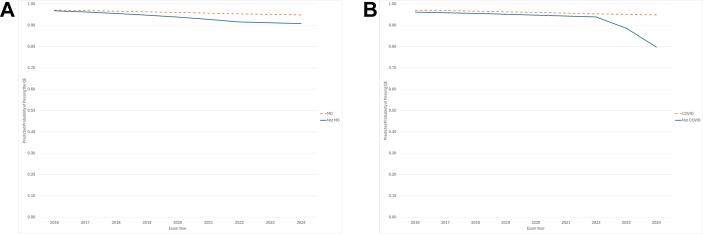
A, Notable changes to the predicted trajectories of QE outcome and the interaction of slope 2016-2022 and medical-degree type. B, Notable changes to the predicted trajectories of QE outcome and the interaction of slope 2022-2024 and training during COVID.

Two significant interactions remained in the final model: degree type with time slope 2016-2022 and training during the COVID pandemic with time slope 2022-2024. Although the main effect of training during the COVID pandemic was not significant, it was retained in the final model due to the significant interaction between COVID and time slope 2022-2024. To help interpret these results, the interactions were plotted against the predicted probability of passing the QE over time. The interaction between degree type and slope 2016-2022 indicated the predicted probability of passing the QE differentially changed from 2016 to 2022 by degree type, wherein MD graduates had a lesser decline over time than non-MD graduates (Fig 2A). Additionally, the interaction between training during the COVID pandemic and time 2022-2024 was significant, suggesting the predicted QE pass rates between 2022 and 2024 declined at a lower rate for physicians who trained during the pandemic vs those who did not (Fig 2B).

## 4 Limitations

Several limitations merit consideration. First, as a retrospective study, the findings are subject to unmeasured confounders. There are limited data on program-specific factors over time (eg, changes in total faculty, faculty support), as well as select applicant factors (eg, parental income, extension in training, and additional demographic variables). Additionally, the EM Model is updated every 3 years, most recently in 2022. Updated EM Models are used to develop the QE 2 years postpublication to allow the field to respond to changes in content. Thus, 2024 was the first test based on a new model and it is too soon to know if this will further impact outcomes. Although the QE is psychometrically equated across years and topics are mapped to the EM Model, it is possible that training may lag behind the topics, resulting in declines due to inadequate exposure to newer topics. Standard setting occurs every 5 to 7 years and is a systematic and judgmental process conducted by a panel of clinical professionals and measurement experts to determine a new passing score for an assessment. This occurred most recently in 2019, and it is unclear to what degree this may have influenced the pass rate inflection points. Moreover, we had limited program-level data and were not able to fully account for the influence of program-specific factors on QE pass rates. Finally, the most recent changepoint was 2022, limiting the ability to assess factors and the continued trajectory beyond 3 years. Future research is needed to further evaluate this over longer periods of time.

## 5 Discussion

This study provides the most comprehensive analysis to date of longitudinal trends in performance on the ABEM QE and identifies key inflection points and associated factors contributing to the recent decline in pass rates. Our findings confirm that the recent decline in QE performance is not an isolated anomaly but rather represents a statistically significant changepoint occurring in 2022 following previous changepoints in 1988 and 2003. These findings have important implications for EM physicians, residency programs, and board certification processes.

An understanding of the factors associated with each inflection point can provide key insights for each changepoint. The first inflection point was seen in 1988, which began a reversal in pass rates with a sharp rise beginning in 1990. This rise may reflect the first time that EM physicians retook the QE to maintain certification, thus boosting pass rates with more experienced physicians adding to the cohort. This increase may also reflect advances in training as the specialty evolved over time. The second changepoint occurred in 2003, wherein the rise in scores became less pronounced over time. This time period overlapped with when ABEM launched the ConCert recertification examination, thus removing part of the cohort who previously contributed to the scores via the previous QE-based recertification process. Notably, the second major decline in pass rates since the QE first began was seen in 2022. Unlike earlier changepoints, the 2022 inflection point did not experience a sudden shift in re-examination participants nor a new recertification (ConCert) examination, prompting a critical need to further explore this phenomenon.

In analyzing this trend, we identified 2 factors that had an impact on examination scores and were significantly different before and after the 2022 changepoint. The first element was undergraduate medical education, with those receiving an MD degree having a slightly lesser decline in scores prior to 2022. One explanation for this finding may be the more limited options for home EM rotations among DO students, resulting in fewer mentorship and tailored EM experiences prior to starting residency.^39^^,^^40^ This may highlight the potential need for more EM exposure and dedicated advisors for students graduating from osteopathic medical schools, particularly given the more recent rise in DO applicants matching into EM residency programs.^41^ Although a performance gap remains, the sharper decline of DO students ended after the 2022 inflection point.

The other major element was the COVID-19 pandemic. COVID created a marked disruption to the clinical environment as well as the training experience of learners.^17^, ^18^, ^19^ Interestingly, we found that those who trained during COVID experienced a lesser decline in QE pass rates between 2022 and 2024 than those who trained pre-COVID. This higher pass rate may reflect increased opportunities for studying due to reduced patient exposure or enhanced access to a wider range of educational experts through virtual didactics.^42^, ^43^, ^44^ Notably, the effect on QE pass rates among students who completed their medical school training during COVID remains unclear and future longitudinal work is needed to better understand the influence of COVID on their pass rates.

Several additional factors were found to be related to the decline in QE performance, including length of residency training received, physician race (URiM), country of medical school attendance (IMG/USMG), and performance on the ITE. However, these factors did not exhibit a differential change in slope or intercept before and after the changepoint that was identified, meaning they are not meaningful in terms of understanding what changed before and after the inflection point. Moreover, although these main effects were statistically significant, further examination of each effect revealed that the differences between groups over time were small in magnitude. For example, after controlling for other variables, the predicted probabilities of 3–year residency graduates passing the QE from 2016 to 2024 ranged from 0.96 to 0.98. In contrast, the predicted probability of 4-year graduates passing the QE from 2016 to 2024 ranged from 0.97 to 0.99. When compared, the differences in predicted probabilities between the 2 groups across time ranged from −0.009 to −0.004, indicating 3-year graduates were predicted to have slightly lower probabilities of passing the QE than 4-year graduates between 2016 and 2024. Interestingly, we did not find an independent association with QE pass rates on residency transfer or leave of absence. Although these can often reflect either lack of institutional support or personal factors, it does not appear that these influenced QE pass rates and should be reassuring to those learners who opt to either transfer or take a personal leave of absence.

However, our findings suggest that broader systemic issues are also likely contributing to the decline in QE pass rates. Faculty shortages, health system financial pressures, and rising service demands can erode individualized curricula or mentorship historically available in residency programs. These environmental constraints may reduce the opportunities for deliberate practice and feedback critical for clinical reasoning development and examination readiness.^45^^,^^46^

The implications of these findings demonstrate the need for robust, multimodal interventions to support learners and reverse the current trend in declining QE pass rates. This work should begin in the medical school environment, ensuring students have robust access to EM mentorship and clinical opportunities that emphasize building a sound foundation of medical knowledge and diagnostic reasoning skills. Residency programs should also monitor and support at-risk learners, ensuring there are dedicated resources and focused remediation strategies in place early on to help foster their academic success. Reflective learning models such as that elaborated in the master adaptive learner can help learners to build their adaptive learning skillsets to succeed outside of the medical school classroom.^47^, ^48^, ^49^, ^50^ Moreover, institutions should invest in faculty development, ensure sufficient protected time, and combat burnout among learners and faculty to ensure the resilience of programs and their leadership. Future research should examine whether any extension in training has an impact on QE pass rates.

This study highlights a key shift in QE pass rates among EM physicians and contextualizes this shift within the 3 primary changepoints since the QE was introduced. The recent decline in current QE pass rates reflects a complex interplay of multiple factors, with our model suggesting a relationship between medical school degree type and COVID pandemic training. This finding emphasizes the need for future research to better understand potential contributing factors. These may also inform multimodal structured interventions to address these challenges to support the success of future trainees and protect the integrity of board certification.

## Author Contributions

Study concept and design: SRW, MG, FKA, MAB, YC, SEF, DLG, LMH, LH, OO, MMJ, and KBJ (all authors)

Acquisition of the data: KBJ and MMJ.

Analysis and interpretation of the data: KBJ and MMJ.

Drafting the manuscript: SRW, MG, FKA, MAB, YC, SEF, DLG, LMH, LH, OO, MMJ, and KBJ.

Critical revision of the manuscript for important intellectual content: SRW, MG, FKA, MAB, YC, SEF, DLG, LMH, LH, OO, MMJ, and KBJ.

Statistical expertise: MMJ and KJB.

Obtained funding: N/A

Administrative, technical, or material support: MG, MAB, KBJ, and MMJ.

Study supervision: MG, MMJ, and KJB.

## Funding and Support

By *JACEP* *Open* policy, all authors are required to disclose any and all commercial, financial, and other relationships in any way related to the subject of this article as per ICMJE conflict of interest guidelines (see www.icmje.org). The authors have stated that no such relationships exist.

## Conflict of Interest

Drs Barton, Gottlieb, Johnston, and Joldersma are employees of the American Board of Emergency Medicine (ABEM). Drs White, Ankel, Calderon, Farrell, Gorgas, and Holden serve on the ABEM Board of Directors. ABEM receives revenue from the ITE, the Qualifying Examination, and the Oral Certifying Examination. The other authors have affirmed they have no conflicts of interest to declare.
