# Supplementary material for: Declining Performance on the Qualifying Examination: Modeling of a Potential Inflection Point in Emergency Medicine
Source: J Am Coll Emerg Physicians Open. 2026 Feb 12;7(2):100333. doi: 10.1016/j.acepjo.2026.100333 (PMC12924182; doi:10.1016/j.acepjo.2026.100333)
Supplement: Supplementary material [file mmc1.docx]

**Supplemental Figure S1. Bayes Factor Probability Thresholds**

| Bayes factor | Evidence category |
| --- | --- |
| > 100 | Extreme evidence for H1 |
| 30 - 100 | Very strong evidence for H1 |
| 10 - 30 | Strong evidence for H1 |
| 3 - 10 | Moderate evidence for H1 |
| 1 - 3 | Anecdotal evidence for H1 |
| 1 | No evidence |
| 1/3 - 1 | Anecdotal evidence for H0 |
| 1/10 - 1/3 | Moderate evidence for H0 |
| 1/30 - 1/10 | Strong evidence for H0 |
| 1/100 - 1/30 | Very strong evidence for H0 |
| < 1/100 | Extreme evidence for H0 |

**Supplemental Figure S2. QE Pass Rates from 1980 to 2024**


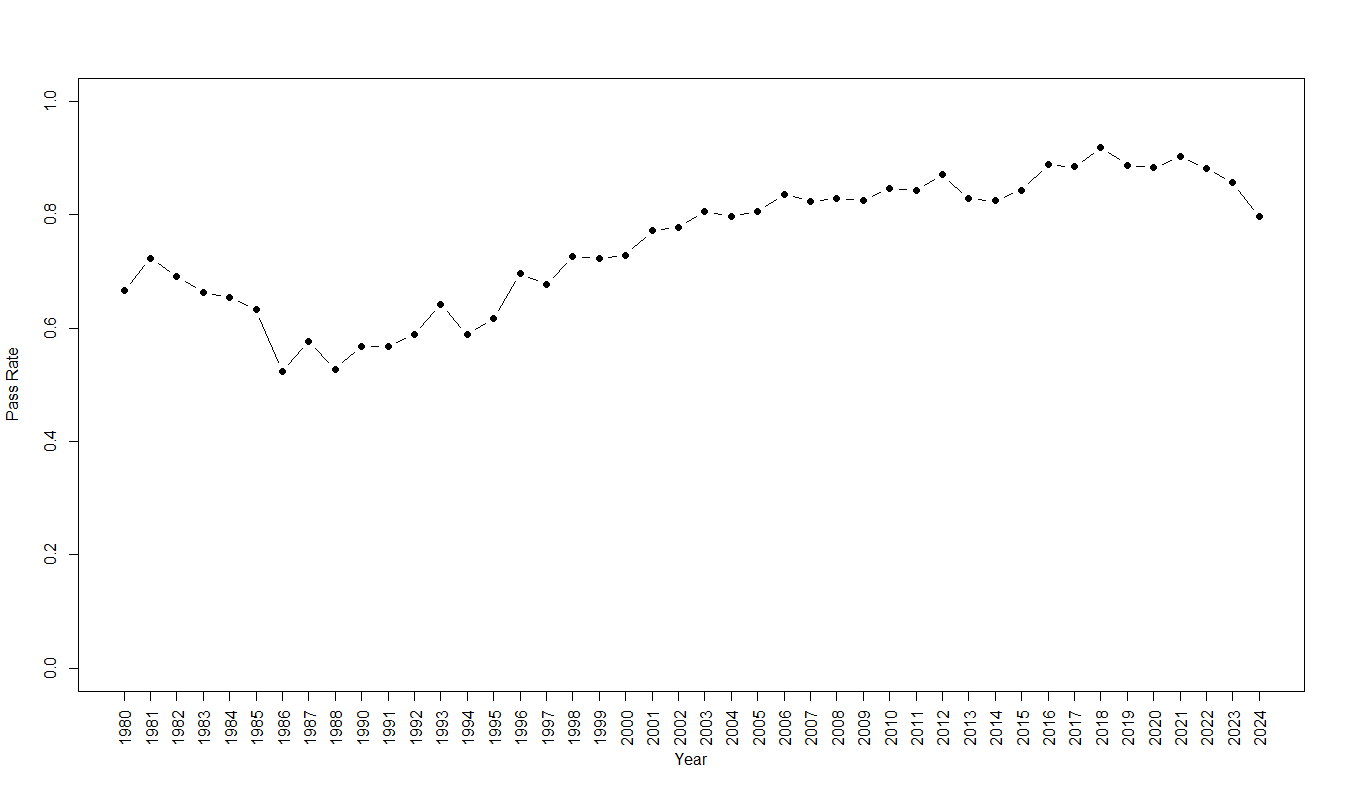


**Supplemental Table S1. Coding scheme for piecewise growth trajectory slopes**

| **Year** | **Slope 2016-2022** | **Slope 2022-2024** |
| --- | --- | --- |
| 2016 | -6 | 0 |
| 2017 | -5 | 0 |
| 2018 | -4 | 0 |
| 2019 | -3 | 0 |
| 2020 | -2 | 0 |
| 2021 | -1 | 0 |
| 2022 | 0 | 0 |
| 2023 | 0 | 1 |
| 2024 | 0 | 2 |

**Supplemental Table S2. Comparison of Three Changepoint Detection Models**

|  | 2-changepoint model | | | | 3-changepoint model | | | | 4-changepoint model | | | |
| --- | --- | --- | --- | --- | --- | --- | --- | --- | --- | --- | --- | --- |
| Coefficient | Est | SE | t | *p* | Est | SE | t | *p* | Est | SE | t | *p* |
| Intercept | 0.74 | 0.02 | 32.90 | < 0.01 | 0.74 | 0.02 | 42.91 | < 0.01 | 0.74 | 0.02 | 38.84 | <0.01 |
| Year | -0.02 | 0.00 | -4.98 | 0.00 | -0.02 | 0.00 | -7.36 | 0.00 | -0.02 | 0.00 | -6.06 | 0.69 |
| DIS 1 | 0.04 | 0.00 | 8.56 | NA | 0.04 | 0.00 | 12.00 | NA | -0.41 | 0.00 | 10.13 | NA |
| DIS 2 | -0.02 | 0.00 | -8.21 | NA | -0.01 | 0.00 | -6.90 | NA | 0.01 | 0.03 | -0.40 | NA |
| DIS 3 |  |  |  |  | -0.05 | 0.02 | -2.87 | NA | 0.00 | 0.03 | 0.02 | NA |
| DIS 4 |  |  |  |  |  |  |  |  | -0.05 | 0.02 | -3.07 | NA |
| Change point 1 | 9.00 | 0.65 |  |  | 9.07 | 0.48 |  |  | 9.00 | 0.55 |  |  |
| Change point 2 | 25.25 | 1.26 |  |  | 23.00 | 1.27 |  |  | 23.00 | 2.01 |  |  |
| Change point 3 |  |  |  |  | 41.64 | 0.60 |  |  | 24.00 | 37.17 |  |  |
| Change point 4 |  |  |  |  |  |  |  |  | 41.85 | 0.51 |  |  |
| Residual Standard Error | 0.03 |  |  |  | 0.02 |  |  |  | 0.02 |  |  |  |
| DF | 38 |  |  |  | 36 |  |  |  | 34 |  |  |  |
| Multiple R-squared | 0.94 |  |  |  | 0.96 |  |  |  | 0.96 |  |  |  |
| Adjusted R-squared | 0.94 |  |  |  | 0.96 |  |  |  | 0.96 |  |  |  |
| *Note: Differences-in-slope (DIS) is an indication of how sharply the slope changes in the segmented regression before and after a given changepoint. Est = estimate; SE = standard error; t = t-value; p = p-value; DF = degrees of freedom.* | | | | | | | | | | | | |

**Supplemental Table S3. Qualifying Examination (QE) pass rates between 2016 and 2024**

| **Year** | **QE Pass Rate** | **Total number of attempts** |
| --- | --- | --- |
| 2016 | 0.89 | 2,119 |
| 2017 | 0.89 | 2,203 |
| 2018 | 0.92 | 2,351 |
| 2019 | 0.89 | 2,503 |
| 2020 | 0.88 | 2,621 |
| 2021 | 0.90 | 2,699 |
| 2022 | 0.88 | 2,876 |
| 2023 | 0.86 | 3,033 |
| 2024 | 0.80 | 3,379 |
